# Cardiovascular risk factors are associated with Idiopathic Normal Pressure Hydrocephalus

**DOI:** 10.1186/2045-8118-12-S1-O41

**Published:** 2015-09-18

**Authors:** Hanna Israelsson, Bo Carlberg, Carsten Wikkelsö, Katarina Laurell, Babar Kahlon, Anders Eklund, Jan Malm

**Affiliations:** 1 Department of Pharmacology and Clinical Neuroscience, Umeå University, Sweden; 2 Department of Public Health and Clinical Medicine, Umeå University, Umeå, Sweden; 3 Institute of Neuroscience and Physiology, Sahlgrenska Academy, University of Gothenburg, Sweden; 4 Department of Neurosurgery, Lund University, Lund, Sweden; 5 Department of Radiation Sciences, Umeå University, Umeå, Sweden

## Introduction

An emerging concept is a possible association between vascular disease/cardiovascular risk factors (CVRF) and idiopathic normal pressure hydrocephalus (INPH). However, the CVRF profile of INPH has not been investigated using a modern epidemiological approach. The objective for this case-control study was to determine the total CVRF profile in a large cohort of shunted INPH-patients, compared to the population.

## Material – method

INPH patients consecutively shunted 2008-2010 in Sweden were scrutinized. Inclusion criteria were: 60-85 years and not having dementia, (i.e., mini mental state examination≥23). Community-based controls were matched to the patients according to age and sex. All participants completed an extensive questionnaire and had a visit to their health-care giver for blood samples, electrocardiogram and anthropometrical measurements. Investigated VRF were: hypertension, hyperlipidemia (measured by apolipoprotein B and A1), diabetes, obesity, psychosocial factors (stress and depression), smoking, dietary pattern, alcohol intake, cardiac disease and physical activity. Cerebrovascular disease (CVD) and peripheral vascular disease (PVD) (claudicatio intermittens, kidney disease (measured by decreased renal function) and stenosis of the extracranial cerebral arteries) were also assessed.

## Results

The study population consisted of 176 INPH patients (mean age 74 years±6SD, 42% females) and 368 controls (mean age 73 years±6SD, 37% females). In the multivariable logistic regression models, hyperlipidemia (OR: 2.380, 95% CI: 1.434-3.950); diabetes (OR: 2.169, 95% CI: 1.195-3.938); obesity (OR: 5.428, 95% CI: 2.502-11.772) and; psychosocial factors (OR: 5.343, 95% CI: 3.219-8.868) were independently associated with INPH. In addition, hypertension (OR: 1.656, 95% CI: 1.017-2.697) and physical inactivity (OR: 2.840, 95% CI: 1.709-4.719) were overrepresented in INPH. CVD were also overrepresented amongst the patients, as well as PVD.

## Conclusion

CVRF are overrepresented amongst INPH patients compared to the population, and CVRF and subsequent vascular disease may contribute to the development of INPH. A more aggressive medical management of CVRF in INPH in addition to shunt surgery is probably needed.

